# Microstructure and Properties of Hollow Octet Nickel Lattice Materials

**DOI:** 10.3390/ma15238417

**Published:** 2022-11-25

**Authors:** Peng Zhao, Deqing Huang, Yongfu Zhang, Hongmei Zhang, Weiwei Chen

**Affiliations:** 1 School of Materials Science and Engineering, Beijing Institute of Technology, Beijing 100081, China; 2 Gaona Aero Material Co., Ltd., Beijing 100081, China; 3 Beijing Key Laboratory of Advanced High Temperature Materials, Central Iron & Steel Research Institute, Beijing 100081, China

**Keywords:** Octet lattice, electrodeposition, structural design, mechanical properties

## Abstract

In this study, electroless nickel plating and electrodeposition were used to deposit thin films on the polymer lattice template prepared by 3D printing, then seven Octet hollow nickel lattice materials with different structural parameters were synthesized by etching process at the expense of the polymer backbone. The microstructure and properties of the Octet structure nickel lattice were characterized by X-ray diffraction, Electron backscattering diffraction and transmission electron microscopy. According to the results, the average grain size of the electrodeposition Ni lattice material was 429 nm, and (001) weak texture was found along the direction of the film deposition. The lattice deformation mode changed with the increase of the lattice length-to-diameter ratio, and it shifted from the lattice deformation layer-by-layer and the overall deformation to the shear deformation in the 45° direction. The strength, modulus and energy absorption properties of the Octet lattice increased with the density, and they were exponentially related to density. In the relative density range of 0.7~5%, Octet hollow Ni lattices with the same density conditions but different structural parameters showed similar compressive strength and elasticity modulus; the energy absorption capacity, however, was weakened as the length-to-diameter ratio increased.

## 1. Introduction

The lattice material is a new porous material that is lightweight, high-performance and multi-function [1,2]. Under the same density conditions, compared with the disordered structures (such as foam and sponges), it has higher strength, modulus, energy absorption capacity and independently designed structural characteristics [3,4,5]. Lattice materials have become one of the research hotspots in aviation, aerospace, shipbuilding, automobile and biomedicine [6,7,8,9,10,11].

Over the past 20 years, with the development of high-precision light-curing 3D printing technology, polymer templates have been prepared by photocuring process, then a film is prepared on it by electron beam deposition, atomic layer deposition, vapor deposition, magnetron sputtering or other processes. Finally, the internal polymer skeleton is removed by sintering or chemical etching to obtain a metal or ceramic hollow lattice [12,13,14,15]. Compared with investment casting, wire weaving and selection laser melting (SLM) and electron beam melting (EBM), hollow lattices prepared by polymer backbone and plating process have the advantages of high precision, ultralight, thin wall thickness and high specific surface area [16,17,18,19,20]. Schaedler et al. used self-propagating photopolymer waveguides (SPPW) and electroless plating processes to prepare a hollow octahedral NiP lattice with a wall thickness of 120 nm and a density of 0.9 mg/cm^3^. It was one of the lightest metal materials at that time all over the world. After being pressed into 50% compression deformation, it was restored to 98% of its original state [21,22]. Valdevit et al. obtained the hollow NiP hollow lattices by changing the content of Ni and P. They studied the properties of octahedral structured crystals and amorphous hollow crystal lattices. Zheng et al. studied the hollow lattice of Octet structure Ni and found that the lattice material had extremely high stiffness. They maintained a close linear proportional relationship between stiffness and density in a wide density range [23]. Meza et al. prepared a hollow Al_2_O_3_ ceramic lattice by a similar process and found that brittle fracture can be inhibited by optimizing the ratio of wall thickness to the diameter [24,25,26].

So far, the research on hollow lattice materials is still in its infancy. Researchers used different manufacturing processes to obtain different components of metal, alloys and ceramic hollow lattices, and the effect of the structure and composition on mechanical properties was studied [15,27,28,29]. They found that the mechanical properties of microlattice materials were affected by topological configuration, structural parameters and properties of skeleton materials [30,31,32,33]. However, the relevant research mainly focuses on the hollow microlattices at the micro and nano scales. With the increase in the single-cell scale, the mechanical behavior and failure mode of hollow lattice materials at larger scales are pretty different from those of micro-lattices [21,23]. The study of the mechanical properties of the large-scale single-cell hollow lattice is still not in-depth enough, affecting the engineering application of hollow lattices. It is urgent to study the influence of the key structural parameters on the mechanical response behavior and failure mode of the lattice.

Among the numerous lattice structures, the Octet lattice structure attracts much attention due to its superior performance. The Octet structure has a regular octahedron as the core, with eight regular tetrahedrons distributed around it. Each of its nodes is connected to 12 rods, all of which are the same length and diameter, and the cube-symmetry of the outer frame, similar to the FCC structure, makes the structure produce an almost isotropic character. Octet has no excess constraints, and it is stable when subjected to force. Its cells are composed of b rods and j nodes, which meet the Maxwell criterion M = b–3 j + 6 ≥ 0; It belongs to the stretch-dominated structure that withstands loads through the stretching or compression of rods and theoretically has a higher mechanical efficiency than bend-dominated structures [34,35].

In this paper, seven different Octet structure Ni hollow lattices were prepared by electrodeposition process. The microscopic structure and structure of hollow nickel lattices were characterized. Through the quasi-static compression test of nickel macrolattice, the influence of the two structural parameters (rod diameter and rod length) on the quasi-static compressive mechanical properties of Octet lattice materials was systematically studied firstly; we believe it will provide an important basis for the application and structural selection of Octet lattice materials.

## 2. Materials and Methods

The preparation process of the hollow lattice was as follows: the 3D model was established using SolidWorks first. The template of lattice polymer was prepared by stereo lithography-based 3D printing method (Lite 600HD, Shanghai UnionTech, Shanghai, China), which method obtained Octet lattice with different structural parameters by curing liquid photosensitive resin by a UV laser beam. The Somos Taurus (Royal DSM, Heerlen, The Netherlands) photosensitive resin was chosen because the carboxyl-containing acrylic ester oligomer is chemically soluble. Then the polymer lattice template was pretreated, electroless plated Ni and electrodeposited in turn. Finally, the hollow Octet structure was obtained by sacrificing the polymer frame.

**Figure 1:**
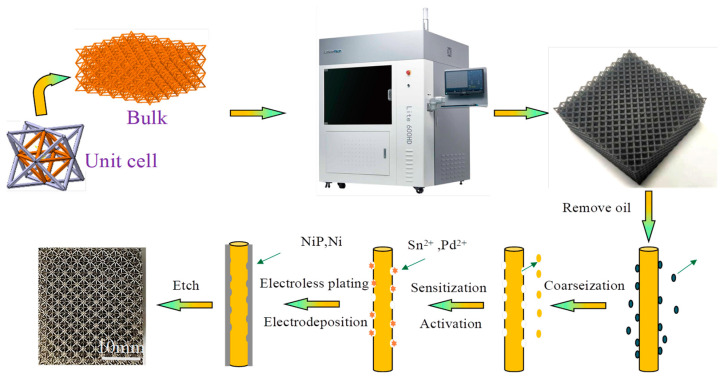
Schematic of the fabrication process for hollow nickel lattices.

To obtain a conductive surface that can be used for electroless Ni plating, the sample was first immersed in an aqueous sodium phosphate solution to remove the oil stain on the surface of the polymer resin, and chromium trioxide solution was used for coarseization. Then it was immersed in a solution containing hydrochloric acid and stannous chloride for sensitization. The template was then immersed in the activator solution to deposit a palladium catalyst using sodium sub-phosphate solution to disperse the colloidal palladium on the polymer template for the purpose of exposing palladium particles as a catalyst. Finally, the sample was immersed in the electroless nickel plating solution, the electroless plating solution used nickel sulfate as the nickel source, sodium hypophosphite as the reducing agent, sodium citrate and ammonium chloride as the complexing agent, and the pH of the electroless nickel plating solution was maintained between 9–10 by adding ammonia at 50 °C. Electroless nickel plating for 30 min was sufficient to deposit a conductive metal layer on the template surface, after which different densities of Octet lattices can be obtained by controlling the electrodeposition time (Figure 1).

The electrodeposition solution was based on commercial nickel sulfonate (AR). To obtain a better structural uniformity, the cathode sample was placed between the two nickel anodes, and electrodeposition was carried out at 50 °C and pH 3.5~4.5. A double pulse power supply was used with a current density of 2.0 A/dm^2^, and the pulse frequency and the duty cycle were set up as 1000 Hz and 1:5, respectively.

After electroless was plated and electrodeposited to the desired thickness, the top and bottom surfaces of each sample were sanded with a grinder to expose the polymer. Then the sample was immersed in an alkaline solution etched to remove the template at 40 °C. The corrosion liquid formula consists of 100 mL ethanol and 100 mL deionization mixed solution, and 10 g sodium hydroxide was added to it.

Scanning electron microscopy (SEM) was used to characterize the morphology of the hollow lattice surface. Electron backscattering diffraction (EBSD) was performed along the thin film deposition direction to obtain information about the grain orientation and grain size distribution of the electrodeposition film. Phase analysis of electrodeposition lattice specimens was conducted by Bruker D8 ADANCE X-ray diffraction (XRD). Information on microstructure was collected using the FEI Talos F200X model transmission electron microscopy (TEM).

The performance of the hollow lattice was affected by the cross-sectional wall thickness-to-diameter ratio (t/d) and truss length-to-diameter ratio (L/d). As t/d increased, the deformation behavior of the octahedral microlattice shifted from Euler buckling to yield [33]. The effect of the length-diameter ratio on the deformation behavior and mechanical properties of macroscopic large Ocete lattice was not clear. In this paper, the hollow lattice with seven structural parameters was designed and prepared. The basic characteristics of the hollow lattice were reflected by the diameter and length of the Octet structural polymer skeleton. Keeping the length of Octet lattice material rod L = 2.5 unchanged, the diameter was adjusted to 0.25 mm, 0.375 mm and 0.5 mm to obtain three hollow lattices. Then kept, the diameter of the lattice material rod constant d = 0.5 mm, and adjusted the length of the rod to 2.5 mm, 3.75 mm, 5 mm and 6.25 mm to obtain another 4 sets of lattices.

The relative density (ρ/ρs) was the ratio of the apparent density of the lattice structure to the density of the matrix material. The density of the lattice material was defined as the ratio of the lattice mass to the volume of geometric space. The relative density of the seven lattice samples prepared was basically similar, all of which were about 3.15%. All sample dimensions are provided in Table 1. According to the data in the article [36], the corresponding value of the solid electrodeposition nickel property were density ρ_s_ = 8.9 g/cm^3^, Young’s modulus E_s_ = 200 GPa and yield strength σ_s_ = 300 MPa.

Finally, the universal experimental machine equipped with video recording equipment was used to test the room-temperature quasi-static compression properties of hollow Ni lattice materials with different diameters and lengths. All specimens for quasi-static compression consist of 4 × 4 × 4 single cells.

## 3. Results

### 3.1. Microstructure

**Figure 2:**
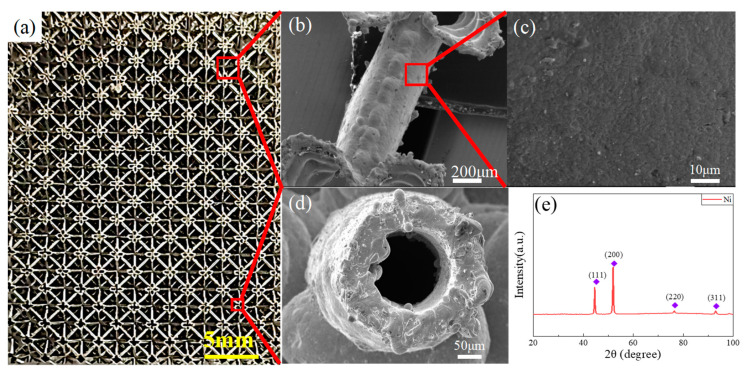
(**a**) Macroscopic pictures of hollow nickel lattices; (**b**–**d**) Hollow lattice surface and cross-sectional topology; (**e**) XRD pattern of electrodeposition film.

Figure 2a showed the Octct electrodeposited hollow nickel lattice sample with a truss diameter of 0.25 mm and length of 2.5 mm, (b) and (c) were the surface topography of the Octet, and (d) showed the orthogonal topography of hollow rod section. As was shown in the figures, the hollow lattice with the polymer removed retained the three-dimensional structure of the polymer template, and the hollow rod interface was circular and nearly circular. The surface of the electrodeposited surface was dense, without obvious holes and cracks. The local nucleation clusters and protrusions appeared on the surface because the position was closer to the anode plate, and the nucleation rate was faster.

The XRD pattern of the electrodeposited samples is shown in Figure 2e. The XRD spectra were consistent with the diffraction standard card JCPDS: 04-085 peak for the nickel metal powder, which has significant diffraction peaks at 44.51°, 51.9° and 76°, and this result corresponded to (111), (200) and (220) crystal faces for Ni, respectively. Compared to XRD standard cards, the Ni lattice XRD map showed a strong (200) diffraction peak, indicating that the crystal plane has produced a selective orientation, possibly due to the presence of impurities in the electrolyte and the electrolyte concentration gradient, that usually reduced the (100) surface energy to increase the intensity of the (100) texture [24,37,38].

**Figure 3:**
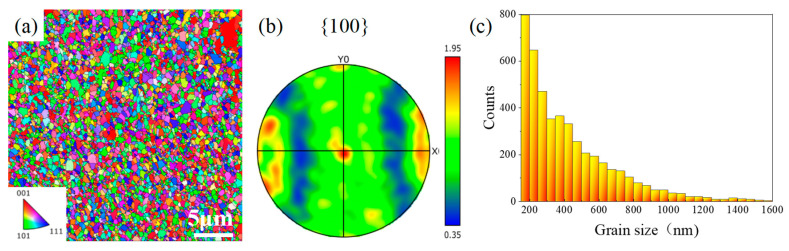
EBSD information of electrodeposition Ni lattice: (**a**) grain orientation imaging figure; (**b**) pole figure image; (**c**) grain size distribution.

An electron backscatter diffraction test was performed cross-sectionally on the lattice sample along the thin film deposition direction. Figure 3a,b show the lattice grain orientation distribution and the pole figure, and a relatively concentrated (001) directional texture can be seen. Small, isoaxial grains in the nanometer scale could be observed in Figure 3a, and the grain distribution was widespread. Figure 3c shows the relationship between the size and the number of Ni grains. These grains were mainly concentrated between 150 and 500 nm, accounting for ~75% of the total grain count, with an average grain size of 429 nm.

**Figure 4:**
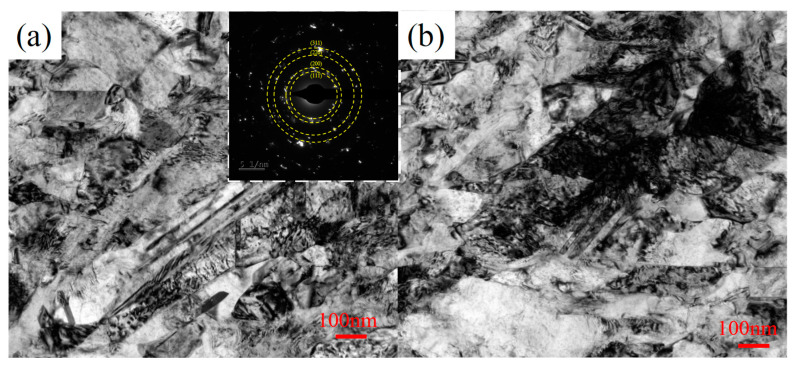
Electrodeposition Ni lattice TEM information: (**a**) brightfield image of microstructure and micro-diffraction spots; (**b**) twins images.

Figure 4 shows a TEM bright-field image of the electrodeposited lattice. It can be found that the particle size of nanocrystalline nickel was not completely uniform; the small particle size was about 10~20 nm, and the large particle size was about 1μm. The sample was a mixed crystal structure of nanocrystalline and microcrystalline, and a small number of large particles with a particle size of several hundred nanometers were embedded in many small particles of tens of nanometers. The electron diffraction pattern of the selected region was annular, and the crystal plane spacing corresponding to the diffraction ring was: 0.203, 0, 0.177, 0.125, 0.107 and 0.102 nm, which belonged to the typical crystal plane of pure nickel. This indicated that the prepared nanocrystalline nickel had a high purity and a face-centered cubic structure.

Grain refinement was an effective means to improve the mechanical properties of nickel metal materials, and nanocrystals can improve the strength and hardness of metal nickel compared with coarse crystals. In addition, small amounts of dislocations and twins were observed inside the nanoparticle crystalline material. The presence of smaller grains and more grain boundaries in nanocrystals hindered the movement of dislocations during plastic deformation, and nanotwins with very fine thicknesses could also increase the strength of the material [39,40].

### 3.2. Deformation Behavior

**Figure 5:**
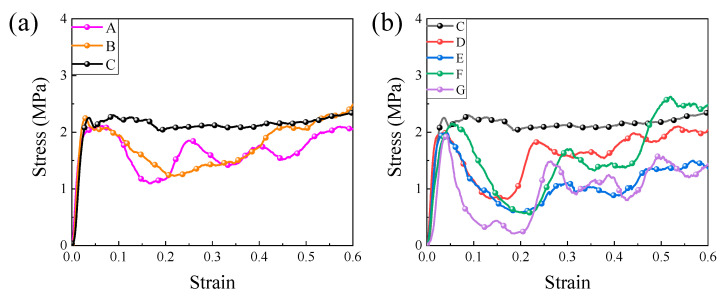
(**a**) Compression curve of lattices of the same length and different diameters; (**b**) Compression curve of lattices of the same diameters and different lengths.

Figure 5 shows the stress–strain curves of quasi-static compression experiments with different length-to-diameter ratio lattices and relative densities of approximately 3.15%. The compression curve of hollow lattice material was mainly divided into three parts: the initial elastic phase, the inelastic phase and the stress plateau phase [41]. When the compressive strain was less than 0.06, the lattice struts were in the elastic deformation region. With the further increase in the compressive strain, the stress–strain curve of the lattice slowly shifted from the elastic region to the platform region, and then the platform region remained within the range of strain of about 0.1~0.6. The next stage of densification usually occurred in the compression of lattice materials, and the stress in this region raised rapidly with the strain until the structure was completely compressed into a solid material. In addition, the stress of the large length-to-diameter ratio remained lower at a longer strain than the lattice with a small length-to-diameter ratio, so the critical strain in the densification region increased as the length-to-diameter ratio increased in the lattice rods. In the figures, the Octet lattice strain was small and did not fully reach the stage of rapid densification.

It can be found that the difference between the stress plateau region and fluctuation region in the hollow lattice curve was caused by the difference in the length-to-diameter ratio of the rod. As the amount of compression deformation gradually increased, the stress–strain curve of the lattice with a large length-to-diameter ratio (e.g., lattice A, E, F and G) was jagged, and yield and plastic deformation occurred when the lattice sample reached peak stress. The stress began to decrease until the first stress underestimation appeared, after which even secondary and tertiary stress rose and fell. Lattices with small length-to-diameter ratios (such as lattice C) had interesting deformation behavior that differed from other lattices, where the lattice reached the stress peak and then a slight decrease, followed by a longer stress plateau region. In the stress plateau phase, the strain gradually increased, but the stress remained basically unchanged. Its compression curve was basically similar to that of typical elastoplastic foam materials.

**Figure 6:**
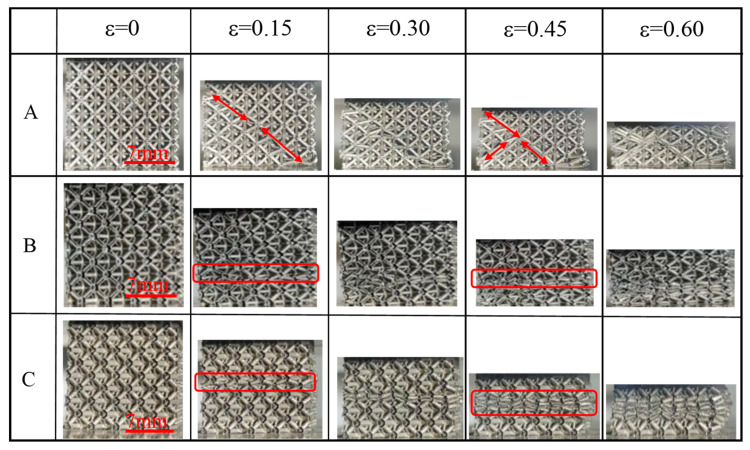
Quasi-static compression deformation process of the lattice with different rod diameters of the same length.

**Figure 7:**
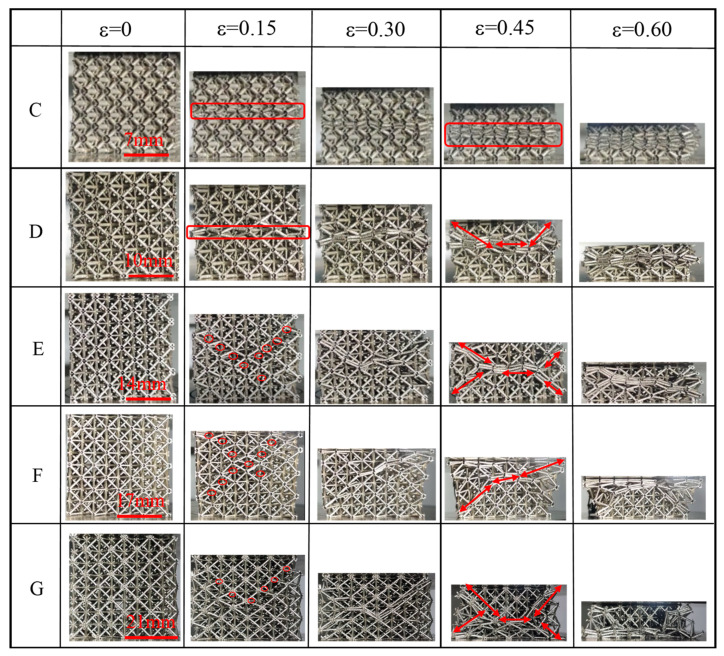
Quasi-static compression deformation process of the lattice with different rod lengths of the same diameters.

Figure 6 and Figure 7 showed the compression deformation process of different diameters and lengths (the relative density was approximately 3.15%). Figure 6 represents the three lattices (with the same length) of A, B, and C in Table 1. Figure 7 referes to the five lattices (with the same diameter) C, D, E, F, and G in Table 1. A common deformation phenomenon can be seen in both figures, that lattices (e.g., lattice B, C, D) with small length-to-diameter ratios (L/d < 10) showed the characteristics of overall deformation and layer-by-layer deformation, the deformation first occurred in the middle layer of the sample and gradually extended towards both ends with marked lateral expansion. This may be due to the increased ability of the struts to transmit axial loads in these short and thick lattice samples. The stress distribution on the truss was relatively uniform, causing basic simultaneous deformation at different positions [42].

As the length-to-diameter ratio increased, the hollow lattice deformation mode shifted from overall deformation and layer-by-layer failure to shear failure in the diagonal direction. When the long-diameter ratio (L/d ≥ 10) lattice (e.g., lattice A, E, F, G) was subjected to axial load, the stress concentration first generated at the node, which caused the rod to rotate around the node to produce plastic deformation even brittle fracture, causing the stress–strain curve to decreased. When the deformation unit of the specimen overlaps the undeformed element, the specimen is re-subjected to the load to raise the curve, and the compression curve decreases again when the deformation or fracture occurs again at the middle node of the structural unit. This failure mainly occurred at the rod node, and there was no obvious deformation in the center of the rod [42,43].

It can also be seen in Figure 7 that the separation of rods and nodes can be observed in the lattice with a length greater than 5 (e.g., lattice E, F and G) in the initial deformation phase. Moreover, the detached point distributed along the 45°direction caused the large length-to-diameter ratio lattice to produce brittle failure at the node. As the amount of deformation increased, the lattice deformation part extended diagonally with the detachment point.

## 4. Discussion

### 4.1. Dependence of Relative Strength on the Relative Density

Compressive strength, elastic modulus and energy absorption were usually three important parameters to evaluate the compressive performance of lightweight materials. The mechanical properties of a lattice structure were usually evaluated as a fraction of the mechanical properties of its matrix material, and they depended on the relative density (ρ/ρ_s_) of the lattice structure. The slope of the initial linear strain curve in the compression test was defined as the elastic modulus E of the lattice material, and the first stress peak was defined as the compressive strength σ of the lattice material. Regardless of the topology, the mechanical properties of the lattice structure were known to decrease as the relative density decreased.

By adjusting the wall thickness of the A–G lattice electrodeposited film, lattices of different relative densities were obtained. Three test samples were intercepted from plates containing multiple cells for repeated experiments, and the test samples came from the same lattice plate. The relative density was basically the same, their mechanical properties were basically the same and the repeatability results were satisfactory.

**Figure 8:**
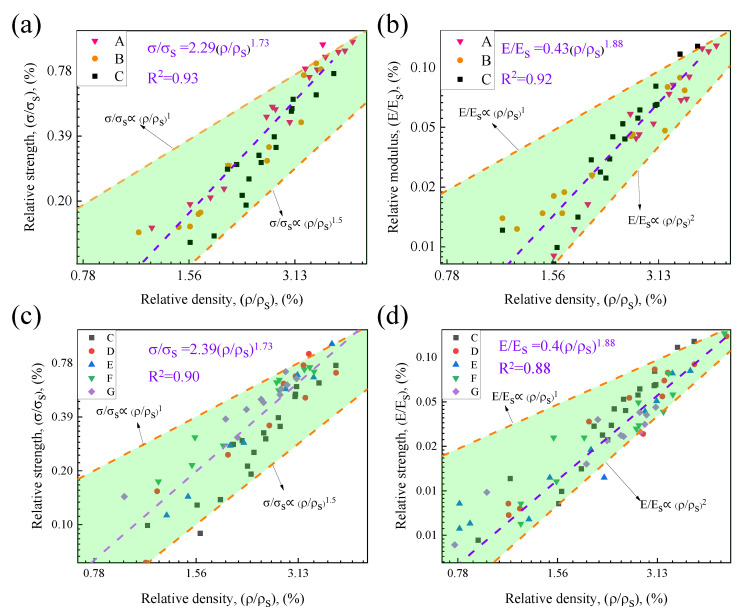
Mechanical properties of lattices with different length-diameter ratios: (**a**,**c**) strength; (**b**,**d**) modulus.

Comparisons were made with the Gibson–Ashby theoretical model to quantify the structural efficiency of Octet hollow lattice samples in this study. Figure 8 shows the relationship between the relative strength and relative density of the Octet lattice for different structural parameters. The strength and modulus of all lattices gradually increased as the density increased. According to the regularity of the data, the exponential relationship can be used to describe the relationship between the relative strength, relative modulus and density of the lattice Ni. Lattices of different rod lengths and rod diameters have substantially comparable strengths and elastic modulus at essentially the same density. The fitting relationships between relative strength, relative modulus and density were σ/σs = K(ρ/ρ_s_)^1.73^ and E/Es = C(ρ/ρ_s_)^1.88^, respectively; all the strength and modulus of lattices of different rod lengths and diameters basically fluctuated around these two curves. The coefficients K and C were generally related to the material configuration. By comparing the mechanical properties and deformation process of different structural parameters of the Octet lattice, it can be found that changing the structure parameters of the Octet hollow lattice changed the deformation characteristics and failure mode of the lattice, but the strength and elastic modulus of the different structural parameters for the Octet hollow lattice did not change substantially [14,21,34,44].

The Octet lattice is a stretch-dominated structure, but by adjusting the length and diameter of the rod, this lattice compression curve in Figure 5 exhibited the generalized stress–strain behavior of two metal lattices dominated by stretching or bending, unlike those mentioned in the literature [25]. In Figure 8, the mechanical properties of the lattice samples were between bend-dominated and stretch-dominated, which did not fully meet the deformation mode of the stretch-dominated structure, but the attenuation rate of the lattice with density behavior was lower than that of the bend-dominated structure. That is probably because there were many structural parameters that affected the strength and stiffness of the hollow lattice. Moreover, parameters such as lattice length, diameter and wall thickness must be studied in detail in order to quantify the influence of structural parameters on mechanical properties.

### 4.2. Dependence of Energy Absorption on Density

**Figure 9:**
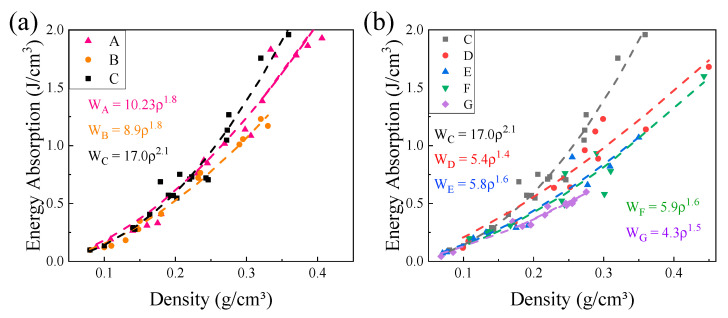
The relationship between the energy absorption performance of Octet hollow lattice and the length-to-diameter ratio and density (**a**) is the same length; (**b**) is a lattice of the same diameter.

The energy absorption can be obtained by calculating the integral area of the stress–strain curve under compressive load, where the energy absorption was determined as:

(1) $$W=\int_{0}^{\varepsilon }\sigma d\varepsilon_{a}$$

where ε was the specified strain, 0.6, and σ was the compressive stress. Figure 9 shows the energy absorption capacity W (0.6) of different Octet lattices at different densities.

Figure 9 shows the change in energy absorption capacity of seven groups of lattice samples with density, and W_A_–W_G_ represented the energy absorption capacity of A–G lattice samples. As can be seen from the Figure 9, the energy absorption capacity increased with density because the rod area under load increased. The lattice energy absorption capacity and density can also be in line with the exponential relationship. The energy absorption capacity gap was small between three groups, A, B and C samples at low density. However, when the density exceeded 0.2 g/cm^3^, the energy absorption of lattice C exceeded A and B. The five groups of different lengths have a clear trend; that was, the energy absorbed by the lattice energy decreases with the increase in length. This showed that reducing the length-to-diameter ratio of the hollow lattice was conducive to improving the energy absorption per unit volume. For example, at a density of about 0.28 g/cm^3^, the energy absorption capacity of sample C was 23% and 45% higher than that of sample B and sample G, respectively. By designing the structure of the Octet hollow truss structure, an ideal energy-absorbing structure can be obtained.

### 4.3. Dependence of Compression Stability of the Density

It can be seen from Figure 5 and Figure 9 that as the lattice length-to-diameter ratio increased, the difference between the stress of the first peak and the stress of the adjacent valley gradually increased, and the stress peak increased when the density increased. Thus, the stability of the stress in the platform region was characterized by the dimensionless parameter θ [18], expressed as θ = ∆σ/σ_peak_. Moreover, ∆σ was the difference between the first maximum compressive strength and the stress of the adjacent valley; the smaller the θ, the higher the lattice compression stability.

**Figure 10:**
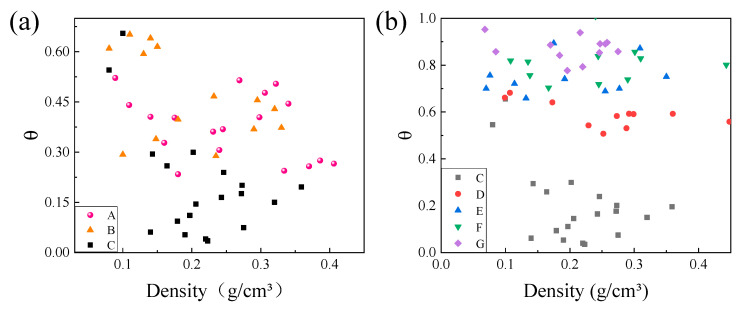
The relationship between compression stability and density of different structural parameters: (**a**) different length; (**b**) diameter diameters.

Figure 10 shows the volatility of different lattice compression curves at different densities. From the figure, it can be found that the value of θ tended to increase with the increase in the lattice length-to-diameter ratio because lattices with a large length-to-diameter ratio were prone to unstable deformation and fracture. The θ of the lattice of C, D, E, F and G five groups remained basically equivalent over a large strain density range, such as the θ value of lattice F is basically equal to 0.8. This lattice collapsed by essentially the same amount after reaching stress peaks at different densities. Moreover, this further caused the energy absorption capacity of the lattice to be lower than that of the small length-diameter ratio. The lattice with a small length-diameter ratio has a longer stress plateau region, which enables the lattice material to have the characteristics of high energy absorption, and it can dissipate the external impact energy through the deformation, collapse, fracture and cell wall friction of the lattice pore.

## 5. Conclusions

In this study, Octet nickel-based lattice materials with different structural parameters were manufactured, and surface morphology and structure characterization of the Ni hollow lattice were carried out. The influence of the structural parameters on the mechanical properties of Octet lattice materials was studied:

(1) The grain sizes of electrodeposition Ni lattice materials were mainly concentrated between 150 and 500 nm, and it had (001) weak texture along the direction of thin film deposition
(2) As the length-to-diameter ratio of the lattice increased, the stress–strain curve volatility of the lattice increased, and the lattice compressive deformation mode shifted from layer-by-layer and overall deformation to shear deformation in the 45° direction.
(3) Hollow Octet lattices with different structural parameters have essentially the same compressive strength and elastic modulus at the same density. The strength and modulus of the lattice increased with the increase of density and belong to the transition region of stretch-dominated and bend-dominated of the Gibson–Ashby model.
(4) The energy absorption capacity and compression stability decreased with the increase of the length-to-diameter ratio of the lattice rod because the macroporous lattice is prone to instability deformation and brittle fracture.

## Figures and Tables

**Table 1:** Summary of architecture and properties of tested microlattices.

Sample	Diameters	Length	Length-to-Diameter Ratio	Relative Density
A	0.25	2.5	10	3.3%
B	0.375	2.5	6.7	3.3%
C	0.5	2.5	5	3.1%
D	0.5	3.75	7.5	3.1%
E	0.5	5	10	3.1%
F	0.5	6.25	12.5	3.3%
G	0.5	7.5	15	3%

## Data Availability

Not applicable.
